# Bioconversion of olive oil pomace by black soldier fly increases eco-efficiency in solid waste stream reduction producing tailored value-added insect meals

**DOI:** 10.1371/journal.pone.0287986

**Published:** 2023-07-21

**Authors:** Olga M. C. C. Ameixa, Marisa Pinho, M. Rosário Domingues, Ana I. Lillebø

**Affiliations:** 1 ECOMARE - Laboratory for Innovation and Sustainability of Marine Biological Resources, CESAM - Centre for Environmental and Marine Studies, Department of Biology, University of Aveiro, Aveiro, Portugal; 2 CESAM - Centre for Environmental and Marine Studies, Department of Chemistry, University of Aveiro, Aveiro, Portugal; 3 Mass Spectrometry Centre, LAQV-REQUIMTE, Department of Chemistry, University of Aveiro, Aveiro, Portugal; Universita degli Studi della Basilicata, ITALY

## Abstract

Olive oil is one of the most important agricultural products in Mediterranean areas, and currently the European Union is the largest producer. Due to technological innovations, Portugal has become one of the main olive oil producing countries over the last few years, accompanied by large amounts of olive oil pomace (OOP), the most representative residue of the olive oil extraction process. This is causing serious waste management problems since current management solutions also present environmental impacts. Here we explored the black soldier fly (*Hermetia illucens*) potential to biotransform OOP into valuable insect meals by feeding them OOP-based diets as substrates. Results show that despite survival rates not being affected by higher replacement (75% and 50%) levels of OOP, there was an increase in larval instar duration. Substrate reduction was significantly lower for higher replacement levels but was not affected up to the 50% replacement level. Feed conversion rate differed among all the treatments, increasing as the replacement level increased, while bioconversion rate, which also differed among all the treatments, decreased as replacement level increased. Differences in larval protein content were only seen at higher replacement levels (75%), with an increase in protein content for replacements of up to 25%. One of the most striking results was the change in fatty acid profile, which became more abundant in monounsaturated fatty acids (mostly oleic acid) as the olive pomace replacement levels increased in comparison with the control substrate, rich in saturated fatty acids (palmitic acid). These results show that BSF can be an effective OOP bioconversion agent, and resulting insect meals can be used as alternatives to currently available saturated fatty acid insect meals.

## Introduction

One of the most important agricultural crops in the Mediterranean biogeographic region is olive orchards (*Olea europaea* L.). The European Union (EU), which produces an average of 2 million tonnes annually and accounts for 69% of global production, is currently the leading producer of olive oil. Spain, which accounts for 63% of the EU’s production, is followed by Italy (17%), Greece (14%), Portugal (5%), and to a lesser extent, France, Slovenia, Croatia, Cyprus, and Malta [1].

The environmental barrier to increasing production along the olive oil supply chain and with the super-intensification of the agricultural phase is the management of the produced by-products (click here to see press releases in Portuguese: https://www.agroportal.pt/sector-do-azeite-esta-a-colapsar-devido-ao-excesso-de-producao-de-azeitona/; https://www.tribunaalentejo.pt/artigos/transformacao-de-bagaco-de-azeitona-passa-ser-controlada-no-alentejo,and law decree 279/2018 setting the legal limitations: https://files.dre.pt/1s/2018/08/16200/0433804338.pdf). Olive oil pomace and its sustainable management are given particular focus in this study.

The two-phase system has become nowadays one of the most popular methods for producing olive oil since the three-phase system, despite having better levels of olive oil recovery, uses a considerable amount of water and generates a significant amount of wastewater that needs to be treated [2]. For every ton of olive oil produced, the two-phase technique generates 4 tonnes of pomace, the thick organic bio-waste known as "olive oil pomace" (OOP) which is made up of fruit pulp, husks, and olive vegetation water [3, 4]. OOP, often known as olive cake or olive husk, is the solid by-product of mechanically extracted olive oil, and since most of it is actually among the most abundant agro-industrial bio-wastes in the Mediterranean region [5]. Due to OOP high phytotoxicity, managing the large amount of bio-waste produced is a challenge for the olive oil industry [6] due to their potential as pollutants in some cases, and to the costs associated with the necessary waste treatments or disposal [7–9]. However, OOP can be used in a variety of ways, including as fertilizer, animal feed (for animals able to digest large amounts of fiber, like ruminants), or as an energy source [10, 11]. Despite the advantages of using OOP as animal feed, there are concerns regarding its safety, palatability, and digestibility [12, 13].

A recent scoping review on the subject identified the primary circular pathways connected to the supply chain for olive oil, concluded that the circular economy pathways (technologies and processes) are still at a low technology readiness level (TRL) and that this can be the momentum to unleash the potential of circular thinking for the olive oil sector, including new circular pathways, along with socioeconomic and environmental challenges [14]. Due to the industry’s current bio-waste management procedures, there are environmental issues such as soil contamination, underground seepage, water body pollution, and foul odour emissions [15]. The main cause of these issues is the wastewater from olive mills, which contains a lot of phenolic compounds. However, due to its influence on three of the primary characteristics of olive mill wastewater—its antibacterial activity, its phytotoxic effect, and its dark colour—it warrants special consideration [16]. The Waste Framework Directive (2008/98/EC), which covers hazardous waste and waste oils, governs bio-waste management in the EU. Preventing waste should come first, followed by reuse, recycling, recovery, and finally disposal. Under the method of recovery, waste is either turned into useful forms or incinerated to recover energy [17]. The Landfill Directive 99/31/EC lays out the appropriate regulations for the final disposal option, landfills. Under certain circumstances, it is the olive mill operator’s responsibility to manage the waste properly until recovery or disposal [18].

Since bioconverter insects can use a variety of substrates and are employed to biotransform wastes or by-products into ingredients with added value, they are potentially capable of processing olive oil pomace as an ecosystem service [19, 20]. One of such bioconversion agents is the detrivorous *Hermetia illucens* (L.), also known as the black soldier fly (Diptera: Stratiomyidae) (BSF).

**Fig 1:**
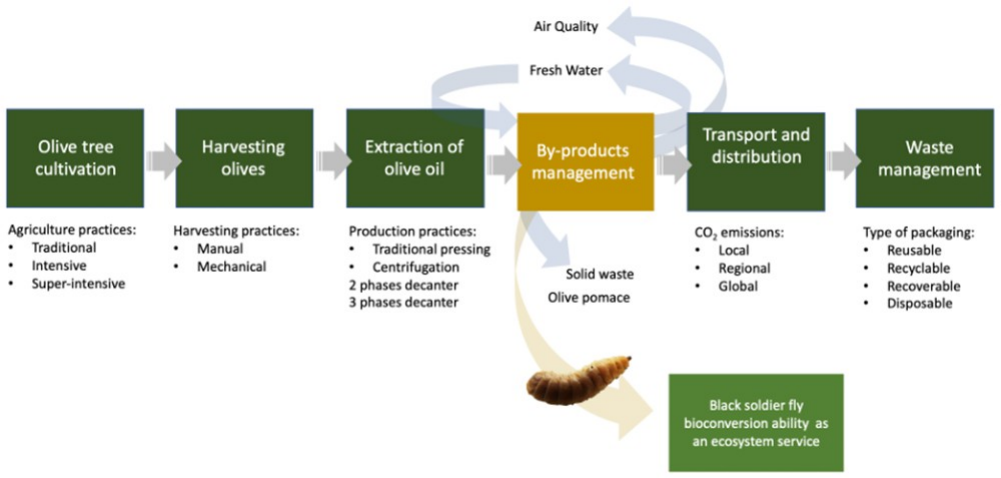
Schematic representation of the olive oil supply chain in an environmental perspective and how the black soldier fly (BSF) bioconversion ability that can be seen as an ecosystem service, fits into the chain.

A schematic representation of the olive oil supply chain from an environmental perspective is illustrated in Fig 1. Having the exponential growth of olive oil production in Portugal as a showcase, we explored the bioconversion ability of BSF and accessed its ability to recover nutrients from olive oil pomace, providing a more sustainable alternative to olive oil pomace disposal. Results are discussed following the patterns of *H*. *illucens* larvae growth and development parameters as well as their bioconversion pattern on lipid molecular species and protein content.

### Portugal as showcase for the need for sustainable production of olive oil

**Fig 2:**
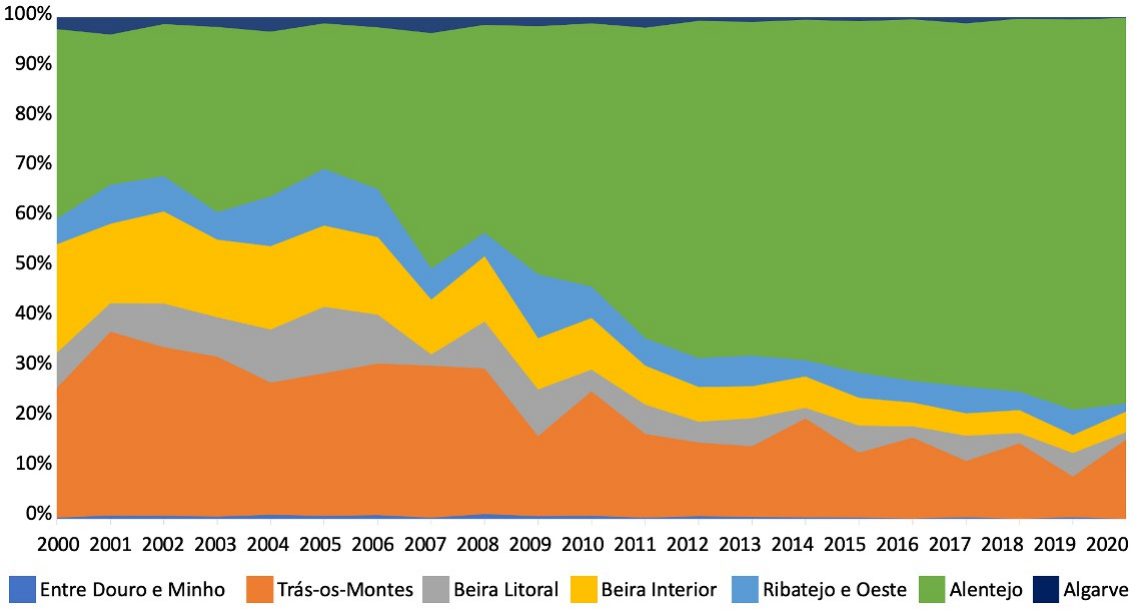
Relative evolution of the average annual olive production in Portugal mainland by region. Metadata from the Portuguese Institute of Statistics [22].

In Portugal, olive oil production has increased exponentially over the last few years, due to technological innovations not only in the field but also at processing factories, making it the eighth largest producing country in the world and the fourth in Europe [21]. Currently, there are 379 444 hectares of olive groves in Portugal, of which 374762 hectares are destined for oil production [22]. The large olive growing regions are located in Trás-os-Montes, Beira Litoral, Beira Interior, Ribatejo and Oeste, and Alentejo, with the latter being currently the main olive oil producing region in Portugal, accounting for 52.2% of the total national area (Fig 2).

The Alentejo region, which was a traditional landscape of cereal steppes and an agro-sylvo-pastoral system locally known as “montado”, has been transformed into a landscape of super-intensive olive groves. However, this increase in the production of olive oil has unfolded several environmental problems, which range not only from the agricultural intensification in the field with the loss of biodiversity and the use of agro-chemicals, but also from the industrial processing of olive oil with the production of bio-wastes. In this region, the current bio-wastes disposal method is incineration. This industrial processing has become a major issue for local populations living close to these factories. For instance, in 2018, the village of Fortes, Ferreira do Alentejo municipality (Portugal), local populations presented a formal complaint to the public ministry [23]. In this document, they reported how their daily lives have become unbearable because they are exposed to smells and fumes impregnated with greasy substances and particles that are expelled from the factory chimneys and are projected to travel more than 30 kilometres. They also reported how their houses and vehicles were covered by an oily residue and ashes, and how the pile of brown dust resulting from the incineration was spread by the wind because it was stored in the open at the factory premises. Furthermore, people living there reported several health issues such as breathing problems, eye inflammation, and burning in the throat, and a villager had medical advice to change residence since he suffered from severe respiratory and lung problems. This has led the villagers to limit their outdoor activities.

For the olive sector in the Alentejo region, this exponential growth in recent years has also brought several challenges to the olive oil supply chain. On the one hand, the storage and management of OOP are currently some of its biggest difficulties due to the structural imbalance because the increase in olive oil production was not accompanied by an increase in the storage and processing capacity of OOP. In fact, due to the environmental problems caused by the incineration of the OOP, there are only four factories in this region, and the establishment of new units was prohibited. Currently, the 2021/2022 campaign expects the production of olive oil to reach values of about 180 thousand tons in Portugal. This campaign is already considered the biggest as long as there are records, and about 900 million kilos of OOP are expected to be produced. This extraordinary production contributed to the total paralysation of all olive grow sector during this period, from the olive picking to the mills that transform it, due to a lack of capacity from these factories to store and process OOP [23]. This bottleneck in the olive oil sector speeds up the need to find short- to long-term sustainable solutions to tackle this environmental, social, and economic problem. Therefore, the Alentejo region in Portugal showcases the urgent need for alternative ways to treat the OOP to maintain a sustainable development of the olive oil supply chain.

## Materials and methods

### Experimental set-up

Black soldier flies used in this study were obtained from a colony housed in our rearing facilities at ECOMARE, University of Aveiro, which has been maintained year-round since 2018 on a control diet consisting of chicken feed and tap water (1:1, v/v) and kept under a 16L:8D photoperiod, at 40±5% relative humidity and 27±3°C.

The trials were conducted under the same conditions in which the stock colony is maintained. Eggs laid in less than 24 hours were collected from the colony and placed in a container to avoid dissection. After hatching, the larvae were fed the control diet referred to above for 6 days before being introduced to the other diets (25%, 50% and 75%).

Each treatment (five replicates per treatment) contained 100 larvae (6 days old, hand counted). On the sixth day, the larvae were harvested according to Bosch et al. [24] recommendations, which included a gentle cleanse (to obtain an accurate weight), after which larvae were weighted, counted into groups of 100 larvae and placed in individual 60 ml containers (with an area of 62.16 cm^2^, which corresponds roughly to 1 larva/cm^2^) with a perforated lid (holes with a 1 mm diameter) to allow air circulation.

### Diet preparation

OOP was kindly supplied from an industrial olive oil extraction unit (NUTRIFARMS, S.A., Ferreira do Alentejo, Portugal), which uses state-of-the-art extraction technology.

Diets using different ratios of OOP (OOP25%, OOP50%, OOP75%) and the control diet (OOP0%), were prepared with chick feed and tap water (1:1, v/v) (see Table 1). These ratios were tested in a previous study conducted by Ramzy et al. [25], however, their OOP was obtained using traditional machines from olive extraction, with fruits produced under a different climatic regime (China), which is known to greatly influence OOP composition [26], which compromises the application of their conclusions in a Mediterranean context. Further, these replacement levels were chosen since a previous study that used OOP 100% replacement level compromised the survival rates [27]. The respective proportions of OOP and dry chicken feed were mixed, aiming for ~63% of moisture, which was achieved by adding tap water, these diets were prepared every two days.

**Table 1:** Diets prepared using different ratios of OOP and control diet.

	Diets			
Ingredients	OOP0%	OOP25%	OOP50%	OOP75%
**Chicken feed**	66.6	49.95	33.3	16.65
**Olive pomace**	0	16.65	33.3	49.95

Subsampling for weighting and feeding was performed every two days. Subsampling was carried out on 10 larvae to not disturb the larvae and affect the conversion efficiency. Added substrate amounts were calculated according to the daily food rate (33.3 mg larva^–1^ day^–1^) and the number of days until the next feeding. This value was chosen after a preliminary test, since individual larva feeding rates can vary according to the type of substrate e.g., particle size, nutritional quality, moisture, and fibre content). Feeding of larvae was continued until 50% of the larvae developed into prepupae, since at higher replacement levels most larvae took a longer time, the prepupae from different treatments were harvested on different days and frozen at -80ºC for further analysis.

### Larval performance parameters

Over the trial larval performance was monitored by periodic weighting of a subsample of 10 larvae, which were previously rinsed with tepid tap water, and dried using kitchen paper. Parameters such as survival rate, number of prepupae and larvae fed on different substrates were also measured at the end of the trial.

### Substrate consumption and reduction efficiency

The efficiency of the BSF to consume and reduce the tested substrates was determined by calculating substrate reduction (1), feed conversion rate (2), and bioconversion rate (3) according to Banks et al. [28] and Diener et al. [29]:

$$Substrate\;reduction\;\left(\%\right)=\frac{total\;feed\;added-residue\;feed\;after\;treatment}{Total\;feed\;added}\;\times 100$$ (1)

$$Feed\;conversion\;rate=\frac{Feed\;added}{Total\;larvae\;biomass}$$ (2)

$$Bioconversion\;rate\;\left(\%\right)=\frac{Total\;larvae\;biomass}{Total\;feed\;added}\;\times 100$$ (3)

### Crude protein

Crude protein content of the tested substrates (before larvae were introduced) and prepupae was calculated from total nitrogen using the nitrogen-to-protein conversion factor. Total nitrogen content was determined using approximately 2 mg/replicate of freeze-dried, ground samples using a CHNS elemental analyser (Leco Truspec Micro CHNS Analyzer Model 630-200-200) and quantified according to Dumas (1831) (ISO 16634–1:2008, n.d.). Crude protein content was calculated using the nitrogen-to-protein conversion factor (Kp) of 6.25. According to [30], this factor is acceptable for estimating the true protein content of most insect species. However, some authors claim that this factor overestimates the protein content, due to the presence of nonprotein nitrogen in insects, for this reason we also report a Kp of 4.76 [31].

### Lipid extraction and quantification

Total lipid extraction was performed according to a modified Bligh and Dyer [32] protocol. Samples were weighed (10 mg, a total of five replicates for each study group) and transferred to glass tubes with Teflon-lined screw caps. Total lipid extraction was performed by adding 1mL of Mili Q-water. Then, the solution of 1:2 (v/v) CH_2_CL_2_: MeOH (3.75 mL) was added, and the mixture was homogenized. After individual homogenization for 2 min using a vortex, samples were incubated on ice on a rocking platform shaker (Stuart Scientific STR6, Bibby, UK) for 30 min. After this procedure, 1.25 mL of CH_2_CL_2_ were added, vortexed for about 1 min after which 1.25 mL of Mili Q-water were added and vortexed for another minute. The mixture was centrifuged at 392× g for 5 min (Selecta JP Mixtasel, Abrera, Barcelona, Spain), and the organic phase was collected to a new glass tube. The remaining biomass residue was re-extracted by adding 1.88 mL CH_2_Cl_2_, vortexed for 1 min, and centrifuged for 5 min. Lipid extracts were collected in the same tube and dried under a nitrogen stream, resuspended in 0.400 mL of CH_2_Cl_2_, vortexed, and transferred to a glass vial, previously weighted. This step was repeated twice to ensure the maximal transfer of the total lipid extract to the vial. The organic solvent was dried under a nitrogen gas stream, and the total lipid extract content was estimated by gravimetry. Lipid extracts were stored at -20ºC, under nitrogen atmosphere until their use in gas chromatography-mass spectrometry (GC–MS).

### Analysis of fatty acid methyl esters

Fatty acid methyl esters (FAME) were prepared from total lipid extracts by alkaline transesterification using a methanolic solution of potassium hydroxide (2.0 M), according to the Aued-Pimentel [33] methodology. Therefore, 1 mL of internal standard (1 μg mL−1 of methyl nonadecanoate (C19:0) in n-hexane) was added to 60 μg of total lipid extract in dichloromethane, followed by 200 μL of potassium hydroxide (2.0 M), prepared in methanol. After 2 min of vortexing, 2 mL of NaCl aqueous solution (10 g L^-1^) was added. The mixture was centrifuged at 392× g for 5 min, and 600 μL of organic phase were collected and dried under a nitrogen gas stream. For GC–MS analysis, the derivatized extract was diluted in 100 μL of hexane. GC–MS data were acquired using an Agilent Technologies 8860 GC System (USA) equipped with a DB-FFAP column with the following specifications: 30 m long, 0.32 mm internal diameter, and 0.25 μm film thickness (123–3232, J&W Scientific, Folsom, CA, USA). The oven temperature was programmed as follows: (1) the initial temperature was set up to 58 ºC for 2 min; (2) a linear increase to 160 ºC at 25 ºC min−1 225; (3) a linear increase at 2 ºC min−1 to 210 ºC; (4) a linear increase at 20 ºC min^-1^ 226 to 225 ºC followed by 20 min at this temperature. The identification of each FA was performed considering the retention times and similarity to MS spectra of FA standards (Supelco 37 Component Fame Mix, Sigma-Aldrich, St. Louis, MO, USA) and available spectra in the Wiley 275 library and the AOCS Lipid Library. The relative amounts of FA were calculated by the percent relative area method with proper normalization using C19:0 as an internal standard, considering the sum of all relative areas of the identified FA. Results were expressed as means ± standard deviation (SD).

### Statistical analysis

Statistical analysis was performed using IBM SPSS Statistics, Version 25 (IBM SPSS Statistics, IBM Corporation, Armonk, NY). Larval performance parameters were compared between treatments by one-way analysis of variance (ANOVA), followed by the Duncan test for post-hoc comparison between different groups. The comparison between groups of OOP control diet replacement was also carried out with a one-way ANOVA, followed, by Tukey’s HSD test for post-hoc comparison. Significance was set at p < 0.05, and all the results are presented as mean ± SD (n = 5).

A chemometric statistical analysis was conducted using MetaboAnalyst (v4.0) to find potential patterns in the lipid molecular species within groups of substrates and relative abundances of different lipid species in *H*. *illucens* larvae fed with the tested substrates. The data was log-transformed followed by auto-scaling to decrease the influence of more or less abundant molecular species (S1 Graphical abstract). Variance in the fatty acids’ profile was accessed by using a Principal Components Analysis (PCA) in MetaboAnalyst, and PCA ordination was constructed in Excel. A hierarchical cluster analysis was carried out using the Euclidean distance similarity measure, and a heatmap using Ward’s linkage was used to construct the dendrogram in MetaboAnalyst. Significant differences were assumed at a critical p-value <0.05.

## Results

### Larval performance parameters

Larval performance parameters are shown in Table 2. There were no differences between the larvae survival rates fed with the different tested diets. However, at the end of the trial, there were more larvae in prepupal stage in diets with lower replacement levels (0–25%), while in diets with higher replacement levels (50–75%) more individuals were in L3-L4 larvae instars.

**Table 2:** Comparison between survival rate, number of prepupae and number of larvae of *Hermetia illucens* larvae fed on different substitution level of OOP at the end of the trial. Different letters represent significant differences.

Substrates	Survival %	Prepupae (number)	Larvae (number)
**0%**	96.20±2.49	71.00^a^±11.38	25.20^a^±12.03
**25%**	96.40±2.41	67.80^a^±7.16	28.60^a^±5.50
**50%**	93.60±1.82	45.20^b^±6.06	46.40^b^±3.29
**75%**	95.20±3.27	7.60^c^±2.51	86.80^c^±3.19

**Fig 3:**
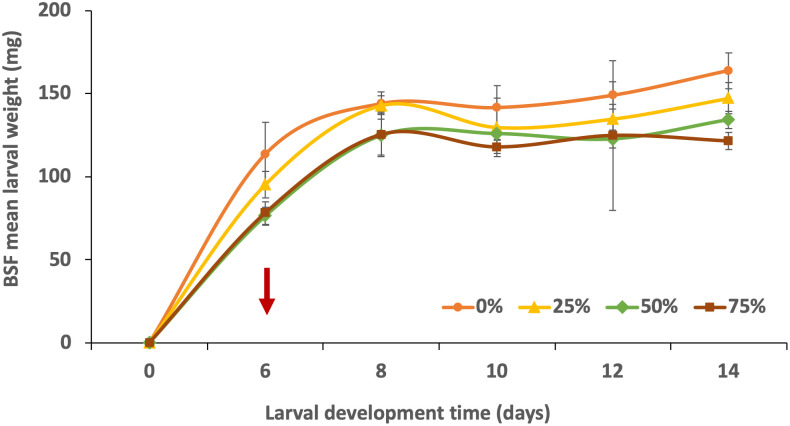
Growth rate of 6-days old *Hermetia illucens* larvae fed with different replacement diets over eight days of feeding trials. Red arrow represents the start of the feeding trial.

Overall, larval average individual weight (Fig 3) increased in all the treatments, but weights were consistently lower in higher replacement diets (50 and 75%).

### Substrate consumption and reduction efficiency

The values of substrate reduction, feed conversion rate, and bioconversion rate are shown in Table 3 (results from the ANOVA are shown in Supplementary material). Regarding substrate reduction, the values ranged between 84.34 and 87.86% for the studied replacement levels, no differences were found between the replacement levels of 0% and 25%, and between 25% and 50%. Substrate reduction was significantly lower at 75% in comparison with other treatments.

**Table 3:** Comparison between substrate reduction, feed conversion rate and bioconversion rate of *Hermetia illucens* larvae fed on different substitution level of olive oil pomace (mean ± SD; n = 5; P<0.05) in wet weight basis. Different letters represent significant differences.

Substrates	Substrate reduction %	Feed conversion rate (FCR)	Bioconversion rate (%)
**0%**	87.86a±1.32	15.38a±0.48	6.50a±0.20
**25%**	86.72ab±0.58	16.30b±0.35	6.14b±0.11
**50%**	85.78b±0.53	18.28c±0.98	5.48c±0.28
**75%**	84.34c±0.76	19.80d±0.37	5.04d±0.09

The feed conversion values ranged between 15.38 and 19.80 and were significantly different between all the treatments, increasing as the replacement level increased, while the bioconversion values, which ranged from 5.04 to 6.50%, also differ among all the treatments, decreasing as the replacement level increased.

### Crude protein and crude lipid

The protein content of the prepupae and the tested substrates (before larvae were introduced) are shown in Table 4. As explained in the methods section, two different conversion factors were calculated for prepupae. Differences in prepupae protein content fed with the tested substrates were only observed for those fed with higher control diet replacement levels (75%).

**Table 4:** Comparison between crude protein (CP 4.76 and 6.25) and crude lipid (CL) content of black soldier fly and tested substrates before larvae being introduced with different replacement level of OOP. Different letters represent significant differences (mean ± SD; n = 5; P<0.05).

Substrates	CP Larvae (Kp = 6.25)	CP Larvae (Kp = 4.76)	CP Substrate (Kp = 6.25)	CL Larvae	CL Substrate
**0%**	41.95^a^±2.05	31.95^a^±1.57	30.25^a^±1.45	38.50±7.36	3.61^a^±0.95
**25%**	43.13^a^±1.50	32.85^a^±1.14	22.24^b^±2.24	34.70±3.69	2.34^b^±0.49
**50%**	41.49^a^±2.09	31.60^a^±1.59	16.22^c^±3.21	35.40±1.98	2.54^b^±0.96
**75%**	37.26^b^±3.15	28.38^b^±2.40	11.42^d^±3.26	35.48±2.49	4.77^c^±0.44

A similar trend was observed in the protein content of the tested substrates as the amount of control feed was replaced by higher amounts of OOP (50 and 75%).

Regarding the crude lipid content of larvae fed with different substrates, no significant differences were observed. However, the crude lipid content of the tested substrates before the larvae were introduced presented significant differences, with exceptions between replacement treatments of 25 and 50%.

### Fatty acid composition

Concerning the larval fatty acid (FA) composition (Table 5), higher amounts of saturated fatty acids (SFA) were observed, and among these, palmitic acid (16:0) was the most abundant, followed by lauric acid (12:0). Among the monounsaturated FA (MUFA), the most abundant was oleic acid (18:1), while for the polyunsaturated FA (PUFA), it was linoleic acid (18:2, n-6), followed by linolenic acid (18:3, n-3), and at the higher replacement levels (>25%), docosadienoic acid (22:2, n-6). Comparisons between the different replacement treatments reveal that there were significant differences among each FA, mostly at higher replacement levels.

**Table 5:** Fatty acid profile of *Hermetia illucens* lipid extracts from four study groups for larvae (L) and tested substrates (S): 0% (control group), 25%, 50%, and 75% replacement of control diet by OOP, expressed as relative abundances (%). Each value is the mean of five samples ± standard deviation (SD). Mean values of the same row (larvae) with different letters indicate significant differences (p<0.05), n.d. = not determined.

Relative abundance (%) ± SD								
	Larvae				Feed substrates (before larvae being introduced)			
Fatty acids	L0%	L25%	L50%	L75%	S0%	S25%	S50%	S75%
**C12:0**	14.00±5.95^a^	13.66±7.38^a^	8.74± 4.79^a^^b^	3.37 ± 1.64^b^	n.d.	n.d.	n.d.	n.d.
**C14:0**	9.07±1.99^a^	8.11±0.76^a^^b^	4.93 ± 0.55^c^	1.96 ± 0.26^d^	0.45 ±0.02	n.d.	n.d.	n.d.
**C16:0**	20.99±2.37^a^	19.48 ± 1.53^a^^b^	16.97 ± 1.39^c^	13.14 ± 0.63^d^	17.01 ±0.45	16.15 ±0.61	16.96 ±1.47	16.64 ±1.21
**C16:1**	6.30±0.75^a^	4.58 ± 0.40^b^	4.22 ± 0.13^b^	4.55 ± 0.48^b^	n.d.	n.d.	0.56± 0.03	0.82 ±0.19
**C18:0**	8.42±1.57^a^	7.07 ± 0.81^a^	5.43 ± 1.63^b^	3.88 ± 0.38^b^	10.06±1.72	8.88 ± 1.63	9.88 ± 1.51	10.32 ± 1.38
**C18:1**	22.98±2.45^a^^b^	30.15 ± 3.80^b^	41.08 ± 3.63^c^	57.07 ± 1.08^d^	28.55 ± 0.82	46.26 ± 2.22	46.43± 3.28	51.95 ± 1.52
**C18:2 *(n-6)***	10.50±1.91^a^^b^	9.49 ± 1.25^b^	11.25 ± 0.67^a^^b^	13.03 ±2.08^a^	41.73 ± 1.58	24.94 ± 0.65	16.97 ± 8.39	17.24 ± 1.31
**C18:3 (*n-3*)**	7.07±1.01^a^	5.48 ± 0.57^b^	5.42 ± 0.65^b^^c^	2.22 ± 1.23^d^	2.20 ± 0.08	1.60± 0.11	7.08 ± 11.52	0.75 ± 0.13
**C20:0**	0.68±0.19^a^	0.39 ± 0.07^b^	0.57 ± 0.15^a^	n.d.	n.d.	0.61 ± 0.08	n.d.	n.d.
**C22:2 (*n-6*)**	n.d.	1.58 ± 0.76^a^	1.39 ± 0.29^a^^c^	0.77 ± 0.25^c^	n.d.	0.67 ± 0.15	0.64 ± 0.15	n.d.
***C22*:*6(n-3)***	n.d.	n.d.	n.d.	n.d.	n.d.	0.90 ± 0.08	1.48 ± 0.29	2.27 ± 0.31
**∑ SFA** ^a^	53.15±12.06	48.71 ± 10.55	36.63± 8.52	22.36 ± 2.91	27.52 ±2.19	25.64 ± 2.33	26.84± 2.97	26.97± 2.59
**∑ MUFAs** ^b^	29.28± 3.20	34.73 ± 4.20	45.30 ± 3.76	61.62 ± 1.56	28.55 ± 0.82	46.26 ±2.22	46.99 ± 3.31	52.77± 1.71
**∑ PUFAs** ^c^	17.57 ± 2.92	16.56 ± 2.59	18.07 ± 1.62	16.02 ± 3.46	43.93 ± 1.65	28.10 ± 1.00	26.18 ±20.35	20.26 ± 1.75
**∑ *n-3***	7.07 ± 1.01	5.48± 0.57	5.42 ± 0.65	2.22 ± 1.23	2.20± 0.08	2.50 ± 0.19	8.56 ± 11.81	3.02 ± 0.44
**∑ *n-6***	10.50± 1.91	11.07 ± 2.01	12.64 ± 0.97	13.80 ± 2.23	41.73 ± 1.58	25.61 ± 0.80	17.62 ±8.54	17.24 ± 1.31
**Ratio (*n-3/n-6*)**	0.67 ± 0.53	0.50 ± 0.29	0.43 ± 0.67	0.16 ± 0.55	0.05 ± 0.05	0.10 ± 0.24	0.49 ±1.38	0.18 ± 0.33

^a^ Saturated Fatty Acids

^b^ Monounsaturated Fatty Acids

^c^ Polyunsaturated Fatty Acids

Regarding the FA composition of feed substrates (before larvae were introduced), they contained mostly monounsaturated oleic acid (18:1), followed by polyunsaturated linoleic acid (18:2, n-6), and saturated palmitic acid (16:0). Lauric acid (12:0) was not found in the substrates, and myristic acid (14:0) was only found in the control in a very low amount.

**Fig 4:**
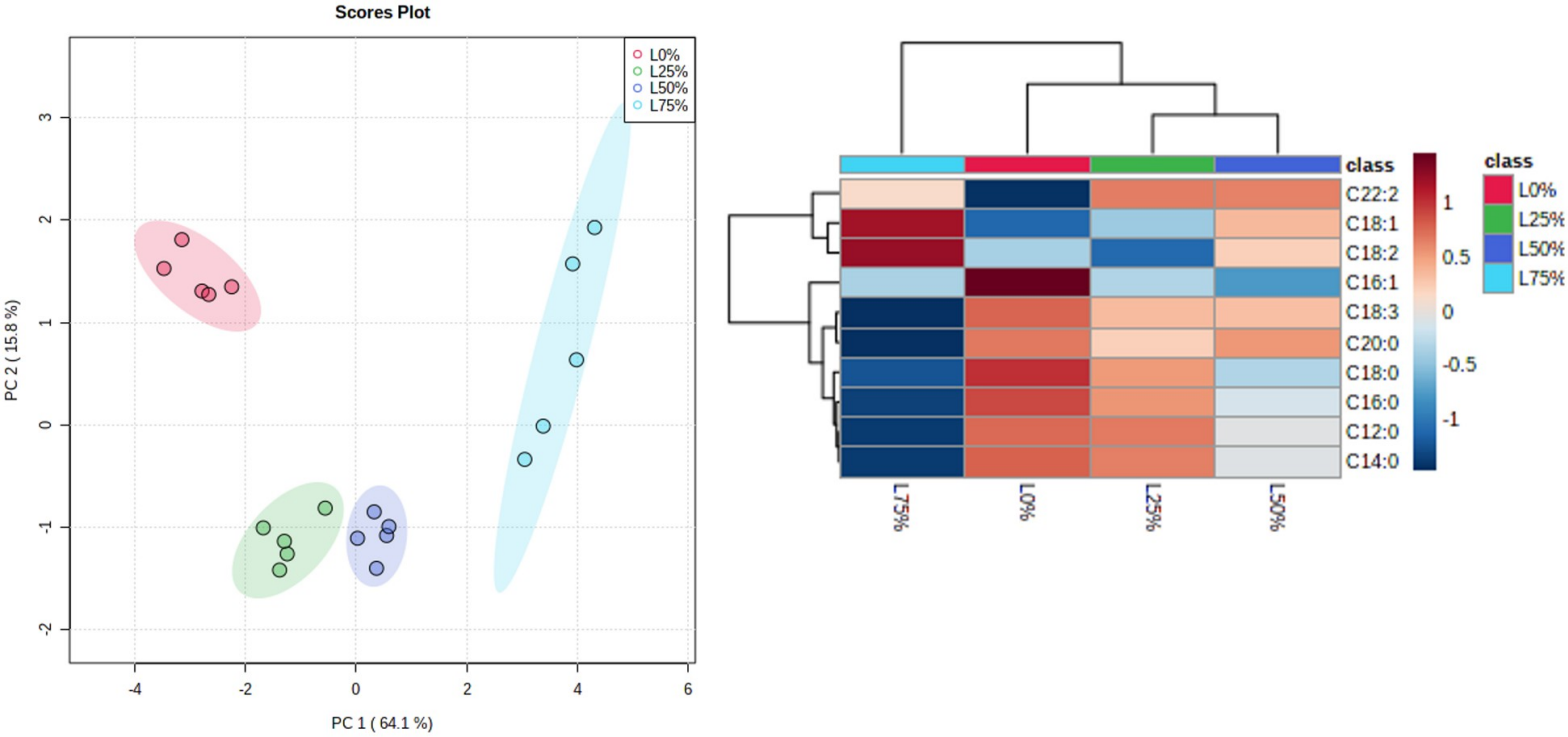
A- Principal component analysis score plot of larvae fatty acid profile of the four study groups: 0% (control group), 25%, 50%, and 75% replacement of control diet by OOP; B—Heatmap showing the correlation of fatty acids and the four study groups: 0% (control group), 25%, 50%, and 75% replacement of control diet by olive oil pomace.

Applying the PCA to the larval FA profile, we extracted five principal components that accounted for 95.5% of the variability for the different diet replacement levels (see S1 Table). From the PCA score plot (Fig 4A), it is possible to see that all samples are clustered into four groups, which shows that the distribution of variability is mainly driven by two principal components that account for 79.9% of the total variance. The correlation between the different FA and feeding groups is shown in Fig 4B where SFA were negatively correlated in L75% and L50% and positively correlated in the control (L0%) and in L25%. MUFAs and PUFAs were positively correlated with L75% and L50% and negatively with control (L0%) and L25%.

## Discussion

One of the main challenges for the current decade concerns sustainable development following the United Nations Sustainable Development Goals (UN SDG’s) for 2030. The SDG’s cover the spheres of natural capital (biosphere), society, and economy, to face global challenges such as climate change, land degradation, a growing population, and limited natural resources. In addition, to overcome these global challenges, the European Green Deal proclaimed goals and targets that highlight economic growth with efficient use of resources and zero net greenhouse gas emissions by 2050 [34]. The European Union is the world’s largest producer of olive oil, and in this context, developing circular bioeconomy strategies for waste management could help to improve resource efficiency in a sustainable way, or eco-efficiency. Managing the enormous amounts of OOP, the most representative by-product of the olive oil extraction process, requires an integrated approach due to the fact that the available solutions have a significant negative impact on the environment. According to ILO [35], there are five drivers of change for value chain development applicable to the olive oil supply chain: system efficiency, product quality, product differentiation, social and environmental standards, and the business environment. This work focuses on environmental standards, supported by the fact that consumers in general are becoming increasingly aware and demanding products that fulfil these requirements. In this context, nature-based sustainable solutions such as the use of insects to biotransform feedstock wastes and industrial organic side streams can be essential in recovering nutrients from wastes, resulting in their reduction and the delivery of value-added feed ingredients with lower environmental impacts under a circular economy approach.

Black soldier fly is currently recognised as a biological tool for reducing biowastes and produce protein. BSF larvae can be used as feed for a variety of animals [19, 36, 37], and their nutritional composition can be tailored by manipulating feed substrates [38]. Increasing demand for novel protein sources, together with the increase in price of both soy meal and fishmeal in the last decade, may have precipitated and contributed to considering insects as a viable ingredient for animal feed [39].

In this work, we explored the potential of the black soldier fly (*Hermetia illucens*) to biotransform OOP into valuable insect meals. Our experience from rearing BSF on OOP was feasible, considering the acceptance of BSF to feed on this substrate (personal observation). Our results showed that despite survival rates not being affected by higher replacement levels of the control diet, there was a trade-off with the duration of the larval stage. A previous study using OOP as a feeding substrate for BSF reported that in addition to negatively affecting larvae growth and development time (no mature larvae were present at the end of the trial in feed substrates that contained OOP), the survival rate was also negatively affected, nevertheless, they did not report any values on this parameter [27]. This may be explained by the fact that they have used 100% OOP without any replacement. In this sense our work provides an indication of a replacement threshold for larvae survival.

The increase in larval instar duration was probably caused by the fact that OOP is a “recalcitrant” by-product due to the presence of tannins and phenolic compounds, which are difficult for insects to biotransform [40]. Despite being shown that insects can, to some degree, detoxify these chemicals [41], this certainly has implications for larval development. In fact, some authors discuss that tannins are not well utilised by BSF and act as antinutritional factors [42, 43]. Further, the presence of recalcitrant fibres such as lignin, cellulose, hemicellulose, and pectin may hamper the ability of BSF to fully digest OOP. For instance, Ramzy [25] showed that high content in fibres increased the development time of pupae (27 days), and BSF larvae did not thrive on 100% OOP due to an increased amount of lignin. However, other studies have previously demonstrated that BSF is able to digest cellulose, hemicellulose, and lignin [44, 45]. Consequently, the presence of these compounds can influence the ability of BSF to reduce this substrate, although in our work, this was not affected up to a 50% replacement level.

The substrate reduction was particularly high (>80%), even at higher replacement levels, showing the great potential of BSF in the degradation of OOP. These values were also higher than those obtained by Ramzy et al. [25] who recorded 19 ± 1.4% in 75% olive pomace replacement, but their olive pomace was obtained using traditional extraction methods and the fruits used were obtained in Chinese olive groves. The protein content of larvae seems to also support these observations since it increased for replacements of up to 25% and significant differences were only observed for higher replacement levels (75%).

One of the most striking results of our work was the shift in larval fatty acid composition, with a general decrease in the amount of SFA (mostly lauric acid) in prepupae and an increase in MUFA content (mostly oleic acid) as the replacement levels of OOP increased. This result confirms that the lipidic composition of black soldier fly can be manipulated by diet. The high amounts of SFA have often been pointed out as a negative feature of the use of BSF in animal feed; despite this, a recent study reported the benefits of its incorporation in fish diets as an immunostimulant, with antiviral properties [46].

Among the SFA, palmitic acid (16:0) was the most abundant, despite not being commonly found. Similar results were obtained by Zulkifli et al. [47] with spray-dried BSF larvae fed with agroindustry by-products. Palmitic acid is the predominant SFA in several marine fish species [48–50], and for other animals such as cows, it has been shown to improve feed efficiency [51] and increase milk yield and milk fat yield [52].

Despite the observed decrease as we increased replacement levels, the other abundant SFA was lauric acid (12:0). Lauric acid is known to have antimicrobial activity, demonstrated against gram positive bacteria and several fungi and viruses [53]. In fact, one of the reasons *Hermetia illucens* gained interest as a novel feed ingredient was precisely due to its high content in lauric acid, which can contribute to animal health, and has the potential to be used as a tool to reduce the use of antibiotics [54]. However, some studies also suggested that the excessive consumption of lauric acid in animal feed can lead to negative effects such as decreased growth and feed intake [55, 56]. Likewise, in fish, some studies suggested that excessive lauric acid consumption can lead to competition with other important fatty acids like omega-3 polyunsaturated fatty acids, which can cause metabolic and physiological disturbances. Moreover, replacing fish oil with vegetable oil blends rich in lauric acid may impact fish health and growth [57]. Some evidence suggests that dietary fibre in animal feed may reduce the absorption of lauric acid in animals [58], which may explain why OPP-fed larvae present a reduced amount of this fatty acid. In this sense, the use of OOP as a feed substrate can limit the advantages of using BSF regarding its content in lauric acid; however, further research is needed to fully understand the specific thresholds at which positive and negative effects may occur.

The increase in MUFA was mostly due to the high amount of monounsaturated FA, oleic acid (18:1). These results were similar to those obtained by Starcevic et al. [27], which also evaluated the OOP FA composition when feeding OOP to BSF. Oleic acid is highly resistant to oxidation and can enhance the activity of antioxidants and antipolymerization agents. In combination with other antioxidants, it can be blended with oils to prevent oxidation [59]. The presence of oleic acid in BSF can be an advantage in preserving animal feeds such as fish feeds, which, due to their high lipidic content are highly susceptible to oxidation, with consequences for feed palatability [60]. This fatty acid has been shown to have hypocholesterolemic effect [61]. In insects, it is the precursor of linoleic acid that is involved in the biosynthesis of prostaglandins and other eicosanoids with various hormone functions in animals [62].

The observed trade-off between lauric acid and oleic acid when increasing OOP levels, can have important implications for animal health and production. Despite the advantages, lauric acid may also have negative effects on animal health, as previously mentioned. On the other hand, adding fat sources high in oleic acid, to animal feed may help improve the quality of animal products, such as meat or milk, by increasing levels of healthy monounsaturated fatty acids. Additionally, diets high in oleic acid may help to improve animal health by reducing inflammation and improving immune function [63].

The advantages of having SFA (mainly short-chain) and MUFA in fish diets are that these are preferable substrates for β-oxidation, aiming at energy production [64], and their dietary supplementation limits the metabolic energy for lipogenesis processes as well as the extension of the oxidation of other fatty acids, such as n-3 PUFA, promoting their accumulation in fish tissues [65].

Regarding the larval composition, the most abundant polyunsaturated FA was linoleic acid (18:2, n-6) this is in line with what was obtained by Starcevic et al. [27] who feed OOP to BSF, and by Zulkifli et al. [47] who feed BSF with non-specified agro-industrial by-products. However, in our work at higher replacement levels (>25%), we also detected the presence of docosadienoic acid (22:2, n-6), which was not found in similar previous studies. Docosadienoic acid (22:2, n-6) is being highlighted regarding its functional properties, namely showing antitumor and antioxidant effects against human breast cancer and strong anti-inflammatory effects [66] and antibacterial properties [67].

In the European Union, besides fish and pet animals, the authorization of insect processed protein use was recently extended to poultry and pig feed formulations, which increases the range of *H*. *illucens* use, since its suitability as a source of protein and lipid for these animals has already largely been demonstrated [68–71].

Regarding protein content, in larvae, differences among treatments were only observed for replacements of 75% for both conversion factors, which is most likely due to the low amount of protein in the substrate. Overall, these results are lower than those obtained by Ramzy et al. [25], but they used wheat bran as a control diet, and the pomace they used was obtained under different environmental conditions, which as previously mentioned influences the nutritional content of OOP.

Regarding crude lipid content, with exceptions for 25 and 50% replacements, there were no differences in crude lipid content in larvae fed with the tested substrates.

Despite the differences, all protein and lipid content of BSF larvae in this study are well within the range reported by previous studies for cultivation of BSF larvae using various vegetable organic wastes 37 to 63% for protein and 30–40% for lipid content [20, 68].

Despite these results, to fully access the sustainability of OOP bioconversion by BSF, the next step should involve a pre-industrial trial followed by its life cycle sustainability assessment (LCSA), i.e., the assessment of the life cycle impact on the environmental, social, and economic components.

## Conclusion

Our work showed that despite the trade-off in BSF lifecycle development, it is possible to use OOP as a feeding substrate with increased eco-efficiency. The most striking result of this work was the change from larvae’s predominant composition in SFA, mostly lauric acid, to more predominant MUFA, mostly oleic acid, showing that by modulating BSF nutritional composition, it is possible to produce tailored value-added insect meals. Nevertheless, there was a trade-off regarding the increase in OOP and reduction of protein content, as well as in the developmental time.

This bioconversion ability can be seen as a regulating service provided by BSF that, in countries such as Portugal, which is expected to become the third-largest olive oil producer by 2030 and where current disposal methods have high environmental/health impacts on local populations, might become a viable solution. Besides the protein content, the resulting insect meals have the potential to contribute to increasing the sustainability of feed industries by recovering valuable nutrients and energy from organic side streams in the olive oil value chain.

To conclude, the bioconversion of OOP by BSF enables the increase of eco-efficiency in solid waste stream reduction to produce tailored value-added insect meals, enabling to target different drivers of change for value chain development.

## Supporting information

S1 Table ANOVA results for the larvae survival rate (%), number of prepupae and number of larvae. (DOCX)

S2 Table ANOVA results from the larvae fitness parameters. (DOCX)

S3 Table ANOVA results for the protein and lipid content of larvae and tested substrates. (DOCX)

S1 Graphical abstract (TIF)
