# Supplementary material for: Bioconversion of olive oil pomace by black soldier fly increases eco-efficiency in solid waste stream reduction producing tailored value-added insect meals
Source: PLoS One. 2023 Jul 21;18(7):e0287986. doi: 10.1371/journal.pone.0287986 (PMC10361471; doi:10.1371/journal.pone.0287986)
Supplement: S1 Table — (DOCX) [file pone.0287986.s001.docx]

**Bioconversion of olive oil pomace by black soldier fly increases eco-efficiency in solid waste stream reduction producing tailored value-added insect meals**

Olga M. C. C. Ameixa, Marisa Pinho, M. Rosário Domingues , Ana I. Lillebø

**Supporting Information**

Table S1 – Anova results for the larvae survival rate (%), number of prepupae and number of larvae

|  | | Sum of Squares | df | Mean Square | F | Sig. |
| --- | --- | --- | --- | --- | --- | --- |
| Survival % | Between Groups | 24.550 | 3 | 8.183 | 1.259 | 0.322 |
|  | Within Groups | 104.000 | 16 | 6.500 |  |  |
|  | Total | 128.550 | 19 |  |  |  |
| Prepupae | Between Groups | 12805.000 | 3 | 4268.333 | 76.322 | 1.07x10^-9^ |
|  | Within Groups | 894.800 | 16 | 55.925 |  |  |
|  | Total | 13699.800 | 19 |  |  |  |
| Larvae | Between Groups | 11989.750 | 3 | 3996.583 | 81.563 | 6.54x10^-10^ |
|  | Within Groups | 784.000 | 16 | 49.000 |  |  |
|  | Total | 12773.750 | 19 |  |  |  |
