# Supplementary material for: Bioconversion of olive oil pomace by black soldier fly increases eco-efficiency in solid waste stream reduction producing tailored value-added insect meals
Source: PLoS One. 2023 Jul 21;18(7):e0287986. doi: 10.1371/journal.pone.0287986 (PMC10361471; doi:10.1371/journal.pone.0287986)
Supplement: S2 Table — (DOCX) [file pone.0287986.s002.docx]

**Bioconversion of olive oil pomace by black soldier fly increases eco-efficiency in solid waste stream reduction producing tailored value-added insect meals**

Olga M. C. C. Ameixa, Marisa Pinho, M. Rosário Domingues , Ana I. Lillebø

**Supporting Information**

Table S2 – Anova results from the larvae fitness parameters

|  | | Sum of Squares | df | Mean Square | F | Sig. |
| --- | --- | --- | --- | --- | --- | --- |
| Substrate reduction % | Between Groups | 33.297 | 3 | 11.099 | 14.974 | 6.62x10^-5^ |
|  | Within Groups | 11.860 | 16 | 0.741 |  |  |
|  | Total | 45.157 | 19 |  |  |  |
| Feed conversion rate (FCR) | Between Groups | 59.092 | 3 | 19.697 | 53.818 | 1.40x10^-8^ |
|  | Within Groups | 5.856 | 16 | 0.366 |  |  |
|  | Total | 64.948 | 19 |  |  |  |
| Bioconversion rate (%) | Between Groups | 6.426 | 3 | 2.142 | 62.087 | 4.94x10^-9^ |
|  | Within Groups | 0.552 | 16 | 0.035 |  |  |
|  | Total | 6.978 | 19 |  |  |  |
