# Supplementary material for: Bioconversion of olive oil pomace by black soldier fly increases eco-efficiency in solid waste stream reduction producing tailored value-added insect meals
Source: PLoS One. 2023 Jul 21;18(7):e0287986. doi: 10.1371/journal.pone.0287986 (PMC10361471; doi:10.1371/journal.pone.0287986)
Supplement: S3 Table — (DOCX) [file pone.0287986.s003.docx]

**Bioconversion of olive oil pomace by black soldier fly increases eco-efficiency in solid waste stream reduction producing tailored value-added insect meals**

Olga M. C. C. Ameixa, Marisa Pinho, M. Rosário Domingues , Ana I. Lillebø

**Supporting Information**

Table S3 – Anova results for the protein and lipid content of larvae and tested substrates

|  | | Sum of Squares | df | Mean Square | F | Sig. |
| --- | --- | --- | --- | --- | --- | --- |
| % Protein in larvae (6.25) | Between Groups | 98.352 | 3 | 32.784 | 6.317 | 0.005 |
|  | Within Groups | 83.035 | 16 | 5.190 |  |  |
|  | Total | 181.388 | 19 |  |  |  |
| % Protein in larvae (4.76) | Between Groups | 57.048 | 3 | 19.016 | 6.317 | 0.005 |
|  | Within Groups | 48.163 | 16 | 3.010 |  |  |
|  | Total | 105.211 | 19 |  |  |  |
| % Protein in substrate (6.25) | Between Groups | 989.661 | 3 | 329.887 | 46.987 | 3.71x10^-8^ |
|  | Within Groups | 112.332 | 16 | 7.021 |  |  |
|  | Total | 1101.993 | 19 |  |  |  |
| % Lipid in larvae | Between Groups | 42.896 | 3 | 14.299 | 0.734 | 0.547 |
|  | Within Groups | 311.491 | 16 | 19.468 |  |  |
|  | Total | 354.387 | 19 |  |  |  |
| % Lipid in substrate | Between Groups | 20.562 | 3 | 6.854 | 16.110 | 4.3x10^-5^ |
|  | Within Groups | 6.807 | 16 | 0.425 |  |  |
|  | Total | 27.369 | 19 |  |  |  |
