# Supplementary figures and images for: Bioconversion of olive oil pomace by black soldier fly increases eco-efficiency in solid waste stream reduction producing tailored value-added insect meals
Source: PLoS One. 2023 Jul 21;18(7):e0287986. doi: 10.1371/journal.pone.0287986 (PMC10361471; doi:10.1371/journal.pone.0287986)

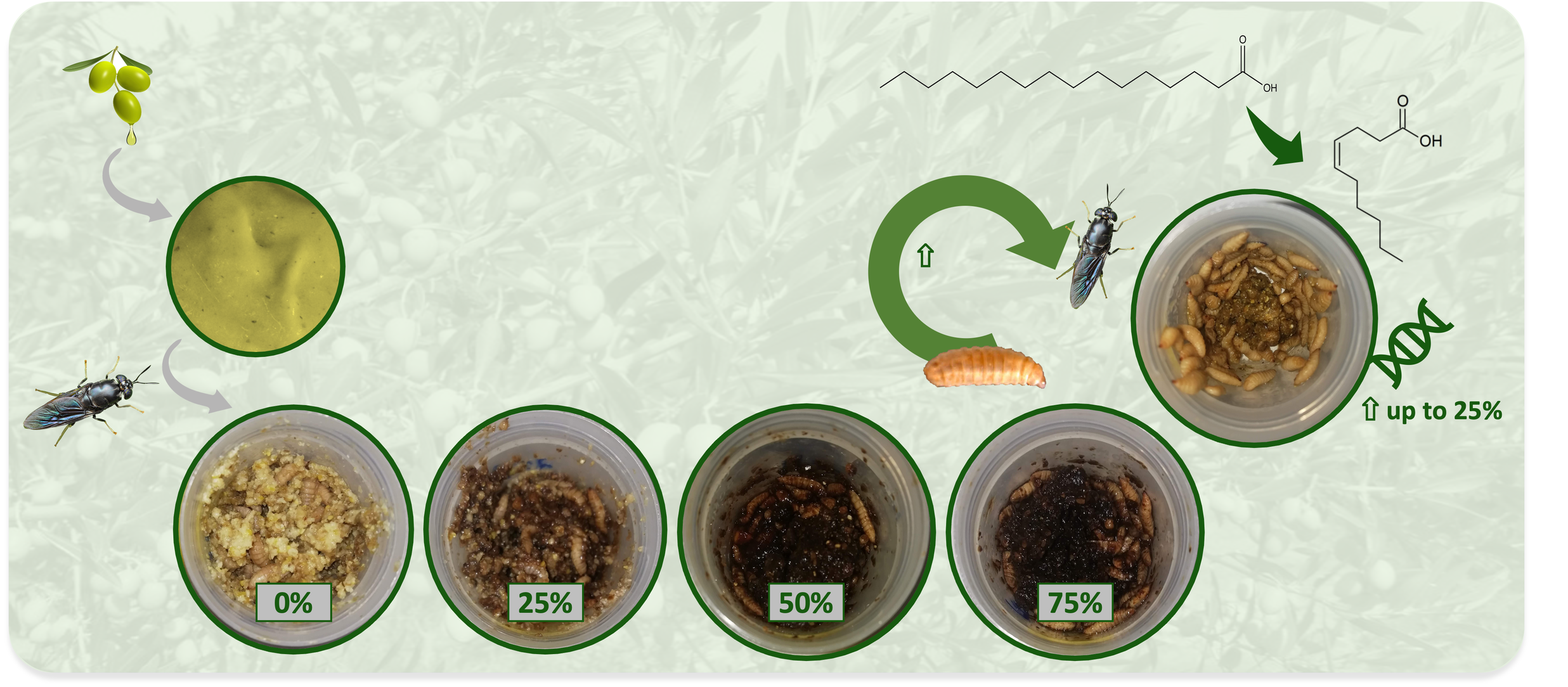

Supplement: S1 Graphical abstract — (TIF) [file pone.0287986.s004.tif]
